# Systematic review and meta-analysis of acupuncture in the treatment of cognitive impairment after stroke

**DOI:** 10.1097/MD.0000000000030461

**Published:** 2022-10-14

**Authors:** Zhen-Zhi Wang, Zhen Sun, Mei-Ling Zhang, Kang Xiong, Feng Zhou

**Affiliations:** a The First Clinical Medical College of Shaanxi University of Traditional Chinese Medicine, Xianyang, China; b Hengyang Medical College, University of South China, Hengyang, China; c The Affiliated Hospital of Shaanxi University of Traditional Chinese Medicine, Xianyang, China.

**Keywords:** acupuncture, cognitive impairment, meta-analysis, stroke, systematic review

## Abstract

We aim to make a systematic evaluation of the clinical efficacy of acupuncture in the treatment of cognitive impairment after stroke, to provide evidence-based medical evidence for clinical practice.

**Methods:**

We searched all the randomized controlled trials of China National Knowledge Infrastructure, Wan fang data knowledge service platform, VIP Chinese periodical service platform full-text Journal Database, Chinese Biomedical Literature Database, Cochrane Library Database, and PubMed Database about acupuncture treatment of post-stroke cognitive impairment (PSCI). Two researchers independently screened the literature and extracted the data according to the inclusion and exclusion criteria. The bias risk assessment manual of Cochrane collaboration Network was used to evaluate the bias risk, and all data were analyzed by Stata16.0.

**Results:**

Fourteen articles were included, with a total of 2402 patients. Meta-analysis showed that acupuncture combined with routine therapy could significantly reduce the score of cognitive impairment symptoms compared with the control group. The mini-mental state examination scale (MMSE) score (weighted mean difference [WMD] = 3.23, 95% confidence interval [CI]: 1.89–4.56, *P* < .01), Montreal cognitive assessment scale (MoCA) score (WMD = 3.41, 95% CI: 0.93–5.89, *P* < .01), Barthel index of activities of daily living (MBI) score (WMD = 4.59, 95% CI: 1.43–7.75, *P* < .01), and Lowenstein assessment scale (LOTCA) score (WMD = 8.60, 95% CI: 6.32–10.89, *P* = .00) were significantly improved in the patients receiving group acupuncture combined with routine therapy.

**Conclusion:**

Acupuncture combined with routine therapy seems to be more effective than conventional therapy alone in the treatment of PSCI. However, the differences between different acupuncture types need to be clarified in more high-quality randomized controlled trials.

## 1. Introduction

Stroke is a common disease that often occurs in people over 50 years old and has the characteristics of high incidence, high mortality, and high disability rate. Its clinical manifestation is transient or permanent brain dysfunction, which occupies the first place in the cause of death of urban residents. Stroke can be divided into 2 categories: ischemic stroke and hemorrhagic stroke.^[1]^ From mild cognitive impairment to severe post-stroke dementia, the mortality, disability, and high nursing costs of post-stroke cognitive impairment (PSCI) increased step by step.^[2,3]^ PSCI has a significant impact on patients, their families, and medical resources. Due to the huge and increasing global burden of stroke, PSCI has become an increasingly serious public health care challenge.^[4]^

Acupuncture is currently recognized as one of the first choices for the treatment of cognitive dysfunction after stroke in China and is often used in combination with other conventional therapies.^[5]^ In addition, acupuncture has been accepted in stroke rehabilitation in many countries, as this treatment is relatively safe and effective in improving post-stroke chronic symptoms, such as disability, dysphagia, and insomnia.^[6–9]^ In the United States, approximately half the stroke survivors engage in some form of acupuncture therapy.^[10]^ Patients with stroke also have a higher TCM utilization rate than people without stroke.^[11]^

According to a modern understanding of acupuncture mechanisms, acupuncture may elicit vegetative reflexes, thereby changing the flow of blood and enhancing the functional properties of connective tissue and organs. Acupuncture signals are recognized as a potent form of sensory stimulation that ascends mainly through the spinal ventrolateral funiculus to the brain.^[12]^ The mechanisms of acupuncture-mediated neuroplasticity have recently attracted increased interest. Accordingly, acupuncture modulation over several cognition- or aging-related gene expressions,^[13]^ plasticity signaling pathways,^[14,15]^ and brain functional connectivities^[16]^ has been studied.

The evidence for acupuncture in clinical practice is currently inconsistent; therefore, the purpose of this systematic review is to analyze the randomized controlled trials of acupuncture in the treatment of PSCI to examine whether conventional treatment combined with acupuncture has the additional benefit and provide an evidence-based medicine basis for the clinical application of acupuncture.

## 2. Materials and Methods

### 2.1. Search strategy

By using the combination of subject words and free words, 4 Chinese databases were searched, including the Chinese periodical full-text database, Wanfang data knowledge service platform, Chinese biomedical literature database, VIP Chinese periodical service platform, PubMed, and Cochrane Library. The retrieval time is from January 1, 2016, to January 10, 2021. English keywords and corresponding Chinese keywords: acupuncture, acupuncture therapy, stroke, infarction, hemorrhage cerebrovascular, cerebrovascular disease, electroacupuncture, electro-acupuncture, cognitive dysfunction, cognitive impairment, cognitive function, etc. No filters are used.

## 3. Literature inclusion and Exclusion criteria

### 3.1. Literature inclusion criteria

We formulated inclusion criteria by the principles of PICOS, and only documents that meet all of the following criteria would be included. Population: clear diagnostic criteria of a stroke complicated with cognitive impairment; intervention: experimental group received routine treatment and acupuncture treatment without restriction on mode and course; comparison: control group received routine treatment; outcome indicators: mini-mental state examination scale (MMSE) (A standardized mental state examination tool for simple screening of temporal orientation, place orientation, immediate memory, attention and computation, delayed memory, language, and visual space),^[17]^ Montreal cognitive assessment scale (MoCA) (An assessment tool for rapid screening of cognitive dysfunction in terms of orientation, short-term memory, executive function, language, abstraction, animal naming, attention, clock-drawing test),^[17]^ Barthel index of activities of daily living (MBI) (A scale used to measure changes in independent living skills such as eating, dressing, continence, and mobility in older people before and after treatment),^[18]^ Lowenstein assessment scale (LOTCA) (A standardized neuropsychological test that assesses orientation, perception, visual motor organization, and mental functioning).^[19]^ Study type: Randomized controlled trials. The language was Chinese or English.

### 3.2. Literature exclusion criteria

Acupuncture therapy was included in the control group; the experimental group did not combine acupuncture therapy with conventional therapy; the course of treatment was <4 weeks; reviews, case reports, guidelines, or, comment; data was incomplete and could not be available.

### 3.3. Literature screening

First of all, duplicate documents were eliminated. Two researchers excluded literature that did not meet the inclusion criteria by reading the title and abstract. The possible literature was read in full text to further judge whether it was included or not. After completion of the preliminary screening, any differences will be resolved through discussion among all authors.

### 3.4. Data extraction

After numbering the final included documents, the data was extracted using the “data extraction Table” designed by Excel, and the extracted contents included: basic information: literature name, author, year of publication, etc. Research characteristics: intervention measures, sample size, course of treatment, outcome index, adverse reaction report, etc.

### 3.5. Document quality evaluation

The literature quality evaluation method evaluates the risk bias of the included literature quality according to the Cochrane systematic evaluation manual, and if there are differences in the evaluation results, it will be discussed and resolved with the third researcher. The evaluation contents include random allocation method, blind method, allocation concealment, data integrity of research results, selective reporting, and other biases. The degree of risk bias, it can be divided into 3 levels: low risk, high risk, and unclear.

### 3.6. Statistical analysis

The data was analyzed by Stata16 software, the weighted mean difference (WMD) was used for the measurement data, and the corresponding confidence interval (CI) of each effect was calculated by 95%. If *P* < .01, the difference was statistically significant. The heterogeneity of the included research was tested, and the corresponding effect model was selected according to the values of the *Q* test and *I*^2^ test. If *P* ≥ .05 and *I*^2^ ≤ 50%, the statistical heterogeneity among the studies is small, then the “fixed effect model” is adopted for the meta-analysis of the curative effect; if *P* < .05, *I*^2^ > 50%, it indicates that the heterogeneity between the studies is large. The “random effect model” was adopted to analyze the curative effect. In addition, the sources of heterogeneity were analyzed by subgroup analysis or sensitivity analysis. We use the funnel chart to detect publication bias, and Begg and Egger tests were used to determine that. When the funnel chart shows a symmetrical inverted triangle and the *P* values of Begg and Egger are >.05, we believe that there is no potential publication bias, otherwise, there may be publication bias.

### 3.7. Ethical approval

Not applicable.

This paper is a kind of quantitative comprehensive evaluation based on literature data of multiple types independent research studies.

## 4. Results

**Figure 1.**
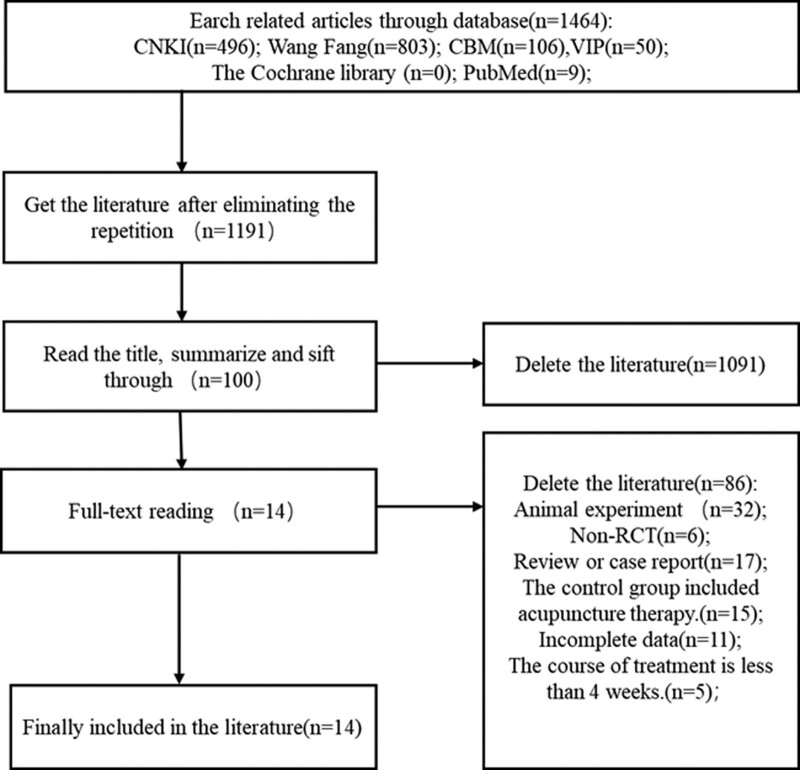
Flow chart of literature screening.

According to the retrieval strategy, 1464 articles were retrieved, 273 repetitive articles were excluded, and 1191 articles were excluded after reading titles/abstracts manually. Re-screening and reading the remaining 100 full-text articles, and finally including 14 articles. The literature screening process and results are shown in Figure 1.

### 4.1. Basic characteristics of the included literature

The basic characteristics of the literature were included in 14 articles, with a total of 2402 patients. The basic characteristics of each study are shown in Table 1.

**Table 1:** The characteristics of included studies.

Study	Ages (yr) (Treatment/control)		Patients no. (Treatment/control)	Intervention (treatment/control)		Retention time	Therapy duration	Outcome measures
Chen, 2020^[20]^	56. 85 ± 17. 12	56. 95 ± 18. 09	40/40	Scalp acupuncture + repetitive transcranial magnetic stimulation	Repetitive transcranial magnetic stimulation	30 min	4 weeks	MMSE/MOCA/MBI
Sha, 2020^[21]^	Unavailable	Unavailable	20/20	Acupuncture + nimodipine	Nimodipine	Unavailable	12 weeks	MOCA
Yang, 2019^[22]^	51.21 ± 7.84	52.14 ± 7.56	30/30	Scalp acupuncture + hyperbaric oxygen	Hyperbaric oxygen	30 min	4 weeks	MMSE/MOCA
Xiang, 2019^[23]^	62.2 ± 4.6	61.9 ± 4.6	40/40	Acupuncture + nimodipine	Nimodipine	Unavailable	8 weeks	MMSE
Gao, 2019^[24]^	Unavailable	Unavailable	43/43	Eye acupuncture + cognitive function training	Cognitive function training	30 min	8 weeks	MMSE/MOCA/LOTCA
Wu, 2019^[25]^	63.5 ± 7.5	64.9 ± 6.8	30/30	Abdominal acupuncture + cognitive rehabilitation training	Cognitive rehabilitation training	30 min	60 days	MOCA/MMSE
Liu, 2019^[26]^	54.31 ± 13.31	55.00 ± 13.84	40/40	Scalp needle + rTMS	rTMS	30 min	30 days	MOCA/MMSE
Yang, 2018^[27]^	68 ± 8	67 ± 8	34/34	Scalp acupuncture + rehabilitation therapy	Rehabilitation therapy	24 h	4 weeks	MMSE/MOCA
Han, 2018^[28]^	60.3 ± 8.1	61.5 ± 8.0	15/15	Scalp needle + rTMS	rTMS	30 min	2 months	MMSE/MBI
Teng, 2018^[29]^	59.47 ± 8.62	60.17 ± 10.68	55/55	Eye acupuncture + routine rehabilitation and computer-assisted cognitive rehabilitation system training	Routine rehabilitation and computer-assisted cognitive rehabilitation system training	30 min	4 weeks	MMSEs/MBI
Zeng, 2018^[30]^	Unavailable	Unavailable	40/40	Jin three needles + cognitive function training	Cognitive function training	30 min	4 weeks	MMSE/MOCA
Jiang, 2018^[31]^	60.15 ± 6.32	58.67 ± 8.03	40/40	Eye acupuncture + computer-assisted cognitive training	Computer-assisted cognitive training	30 min	8 weeks	LOTCA/MBI
Liu, 2017^[32]^	48.93 ± 9.86	46.38 ± 10.35	50/50	Scalp acupuncture + Donepezil Hydrochloride + Cognitive function training	Donepezil hydrochloride + cognitive function training	30 min	3 months	MMSE/MOCA
Xue, 2016^[33]^	63 ± 7	62 ± 8	30/30	Zhi San needle + donepezil hydrochloride + computer-aided Cognitive training	Donepezil Hydrochloride + computer-aided Cognitive training	40 min	4 months	MOCA

LOTCA = Lowenstein assessment scale, MBI = activities of daily living Barthel index, MMSE = mini-mental state scale, MOCA = Montreal cognitive assessment scale, rTMS = transcranial magnetic stimulations.

### 4.2. Quality evaluation

The risk bias was assessed according to the Cochrane guideline. Ten articles^[20–22,24,25,28,29,31–33]^ were grouped by the random number table method, 1 article^[27]^ used the computer random grouping method, and the other 3 articles^[23,26,30]^ did not mention the specific distribution scheme. In the aspect of data collection and evaluation, 1 article^[27]^ mentioned that the implementation of the blind method was single-blind, the other 11 articles did not mention the blind method. In terms of other biases, the diagnostic criteria of 8 articles^[20,21,25,27,29–31,33]^ are not specified, which may affect the authenticity of the study, so they are evaluated as high risk. The quality evaluation of the included literature is shown in Table 2.

**Table 2:** Quality assessment of included studies.

References	Random sequence generation	Allocation concealment	Blinding of participants and personnel	Binding of outcome assessment	Incomplete outcome data	Selective reporting	Other bias
Chen, 2020^[5]^	Low risk	Unclear	Unclear	Unclear	Low risk	Low risk	High risk
Sha, 2020^[6]^	Low risk	Unclear	Unclear	Unclear	Low risk	Low risk	High risk
Yang, 2019^[7]^	Low risk	Unclear	Unclear	Unclear	Low risk	Low risk	Low risk
Xiang, 2019^[8]^	Low risk	Unclear	Unclear	Unclear	Low risk	Low risk	Low risk
Gao, 2019^[9]^	Low risk	Unclear	Unclear	Unclear	Low risk	Low risk	Low risk
Wu, 2019^[10]^	Low risk	Unclear	Unclear	Unclear	Low risk	Low risk	High risk
Liu, 2019^[11]^	Low risk	Unclear	Unclear	Unclear	Low risk	Low risk	Low risk
Yang, 2018^[12]^	Low risk	Unclear	Unclear	Unclear	Low risk	Low risk	High risk
Han, 2018^[13]^	Low risk	Unclear	Unclear	Unclear	Low risk	Low risk	Low risk
Teng, 2018^[14]^	Low risk	Unclear	Unclear	Unclear	Low risk	Low risk	High risk
Zeng, 2018^[15]^	Low risk	Unclear	Unclear	Unclear	Low risk	Low risk	High risk
Jiang, 2018^[16]^	Low risk	Unclear	Unclear	Unclear	Low risk	Low risk	High risk
Liu, 2017^[17]^	Low risk	Unclear	Unclear	Unclear	Low risk	Low risk	Low risk
Xue, 2016^[18]^	Low risk	Unclear	Unclear	Unclear	Low risk	Low risk	High risk

### 4.3. Meta-analysis results

#### 4.3.1.1. MMSE score.

**Figure 2.**
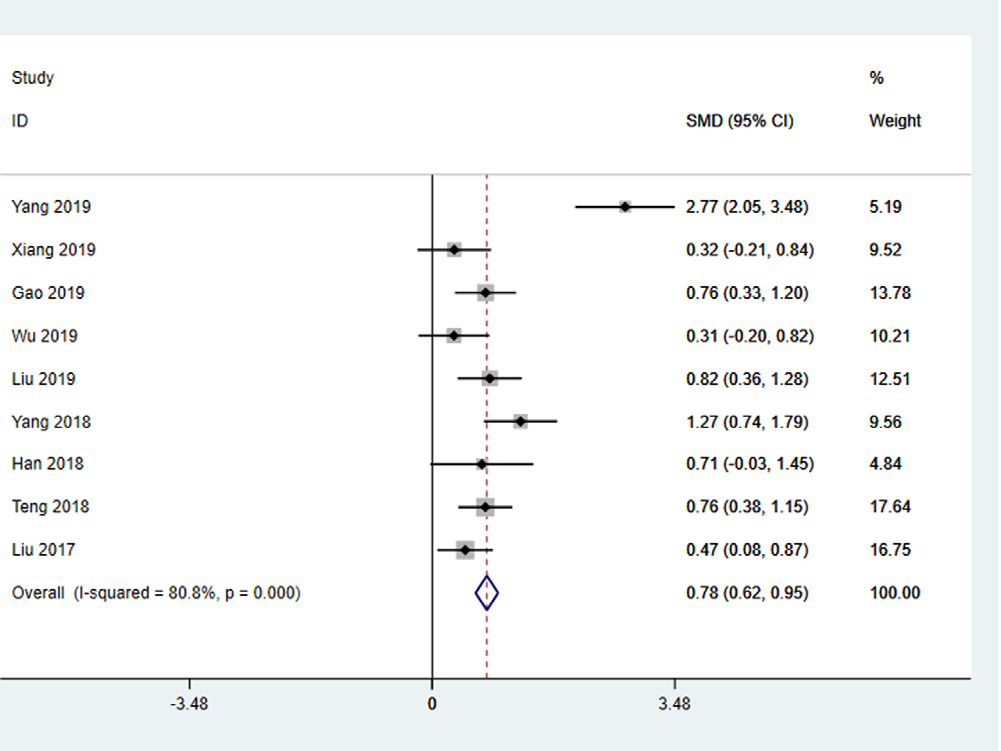
MMSE score of patients with stroke treated with acupuncture. MMSE = mini-mental state scale.

Nine studies^[22–29,32]^ used the MMSE score as the outcome index, and there was statistical heterogeneity among the results (*I*^2^ = 80.8%, *P* < .001), so the random effect model was used for meta-analysis. The results showed that the improvement of MMSE score in the treatment group was better than that in the control group, and the difference was statistically significant (WMD = 3.23, 95% CI: 1.89–4.56, *P* < .01) (Fig. 2). It indicates that the cognitive dysfunction of stroke patients has been significantly improved.

#### 4.3.1.2. MoCA score

**Figure 3.**
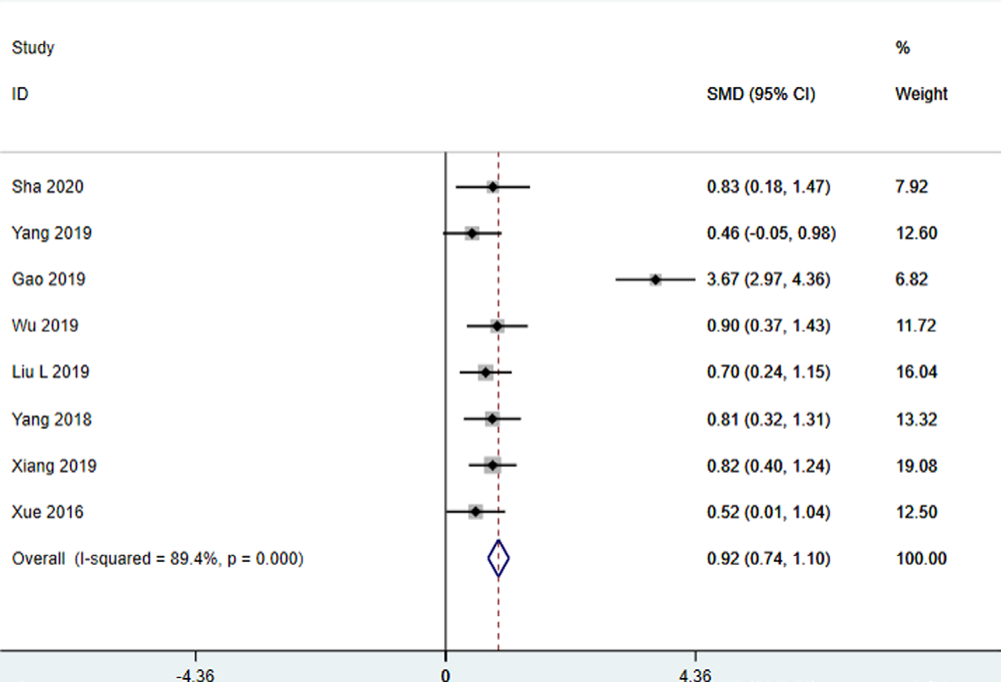
MoCA score of patients with stroke treated with acupuncture. MoCA = Montreal cognitive assessment scale.

Eight studies^[21–27,33]^ used the MoCA score as the outcome index, and there was statistical heterogeneity among the results (*I*^2^ = 95.8%, *P* < .001), so the random effect model was used for meta-analysis. The results showed that the intervention group was superior to the control group in improving the MoCA score, and the difference was statistically significant (WMD = 3.41, 95% CI: 0.93–5.89, *P* < .01) (Fig. 3). It indicates that the cognitive function of patients with mild cognitive impairment has been significantly improved.

#### 4.3.1.3. MBI score

**Figure 4.**
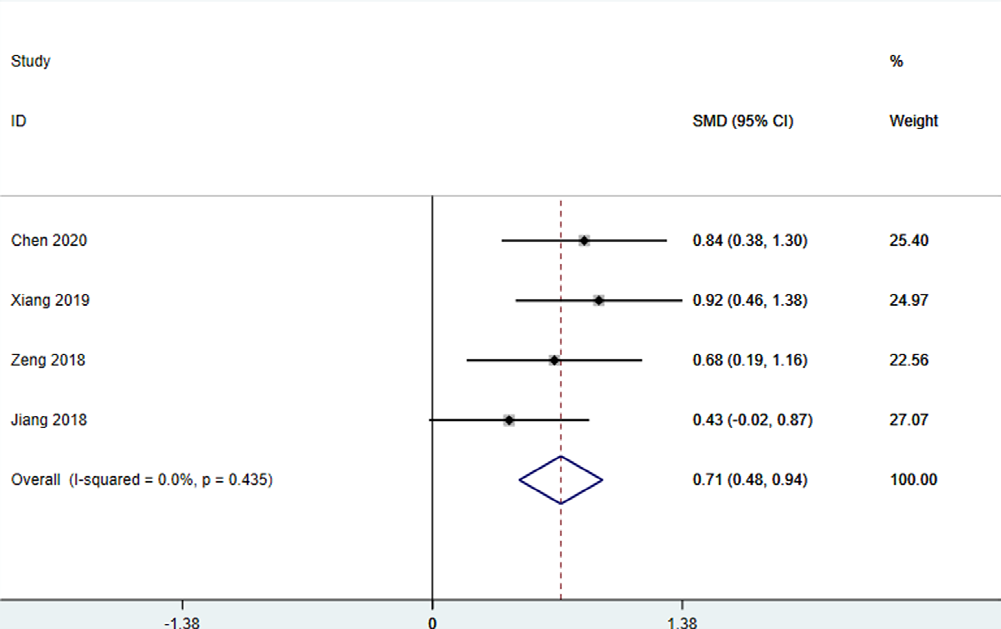
MBI score of patients with stroke treated with acupuncture. MBI = activities of daily living Barthel index.

Four studies^[20,23,30,31]^ used the MBI score as the outcome index. Heterogeneity analysis showed that there was a large heterogeneity among the studies (*I*^2^ > 50%), so the random effect model was used for Meta-analysis. The results of the combined effect of clinical effective rate showed that there was a significant difference in MBI score between the control group and the treatment group, and the effect value was combined with WMD = 4.59 (95% CI: 1.43–7.75, *P* < .01) (Fig. 4). It shows that the self-care ability of stroke patients is improved obviously, which include Feeding, bathing, grooming, dressing, controlling urine and feces, going to the toilet, transferring beds and chairs, etc.

#### 4.3.1.4. LOTCA score

**Figure 5.**
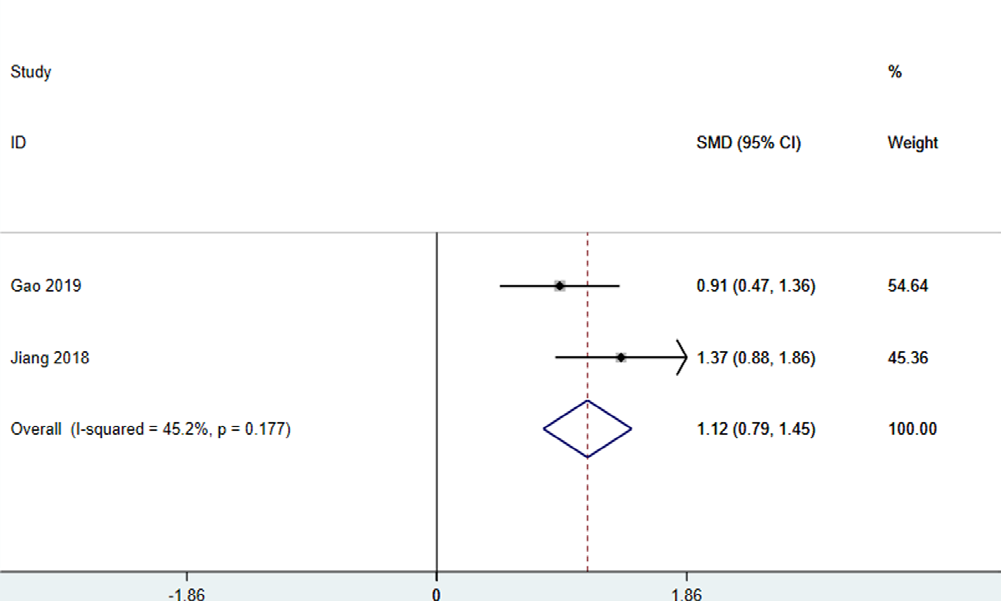
LOTCA score of patients with stroke treated with acupuncture. LOTCA = Lowenstein assessment scale.

Two studies^[24,31]^ used the LOTCA score as the outcome index, and there was no statistical heterogeneity among the studies (*I*^2^ = 0.0%, *P* = .360), so the fixed effect model was used for meta-analysis. The combined effect value of WMD was 8.60 (95% CI: 6.32–10.89, *P* < .01). It shows that the clinical efficacy of scalp acupuncture in the treatment of cognitive impairment after stroke is better than that of the control group (Fig. 5). It showed that the patient’s orientation, perception, visual movement, thinking, and other aspects of the situation improved significantly.

### 4.4. Sensitivity analysis

Sensitivity analysis was conducted by excluding 1 study at a time. We find that the results were robust and did not change the conclusions due to the exclusion of any study.

### 4.5. Publication bias analysis

Because the number of articles included in the MBI score and LOTCA score index groups was <5, it is not suitable for funnel chart analysis. The MMSE score and MoCA score index groups were evaluated for the existence of publication bias. The results showed that although individual subjects deviate from the CI of the funnel diagram, they are roughly symmetrical. In addition, the *P* values of Begg and Egger tests of all indicators were >.05, indicating that there was no potential publication bias.

## 5. Discussion

The 14 studies included in this study included 2402 cases. Meta-analysis results of 4 observational indicators showed that acupuncture combined with other therapies had significant advantages in treating PSCI, improving MMSE score, MoCA score, MBI index, and LOTCA scale compared with other therapies. MMSE score (WMD = 3.23, 95% CI: 1.89–4.56, *P* < .01), MoCA score (WMD = 3.41, 95% CI: 0.93–5.89, *P* < .01), MBI score (WMD = 4.59, 95% CI: 1.43–7.75, *P* < .01) and LOTCA score (WMD = 8.60, 95% CI: 6.32–10.89, *P* < .01). It can be concluded that acupuncture has a good effect on improving cognitive function and promoting the rehabilitation of PSCI patients, which provides a theoretical basis for the therapeutic advantage of acupuncture combined with conventional therapy in treating PSCI.

Traditional acupuncture has a long history in the treatment of stroke, and traditional Chinese medicine has a comprehensive understanding of it.^[19]^ Stroke is also called “dementia” in the Internal Classic of Huangdi. So far, it is considered that “wind, fire, phlegm, qi, blood stasis, deficiency” is its etiology and pathogenesis. ^[34]^ Cognitive impairment often occurs in stroke, vascular diseases, and other diseases. The therapeutic effect of western medicine is limited and the side effects are obvious. Some studies have shown that cognitive impairment belongs to neurodegenerative disease, which is due to the functional degradation of central nervous cells in patients, especially in the cerebral cortex and hippocampus.^[35]^ This leads to cognitive impairment in patients. Personality change, decreased living ability, memory loss and slow response are the common clinical manifestations. After the occurrence of PSCI, the quality of life of patients decreased sharply, and looking for effective ways to improve patients’ cognitive function is of great significance to improve patients’ cognitive function and promote their prognosis.^[36]^

In recent years, acupuncture has been widely used in the clinical treatment of PSCI. Many studies have shown that acupuncture may improve the cognitive function of PSCI patients by regulating enzyme activity, improving synaptic plasticity, and inhibiting the expression of inflammatory cytokines.^[37–39]^ Some studies have found that acupuncture can significantly reduce the incidence of stroke sequelae, has a positive effect on the treatment of cognitive impairment, and reveals that acupuncture can regulate hemorheology and neuron electrophysiology and protect neurons.^[40–42]^ Acupuncture has the characteristics of economy, safety, simplicity, and so on, so it is more and more widely used in clinical practice.

More and more studies have demonstrated the neuroprotective activities of acupuncture therapy. Most of these studies have proposed that acupuncture exerts its therapeutic effects via regulating various signaling pathways. ^[43]^ Such as, some studies have confirmed that acupuncture exerts several beneficial effects on the central nervous system via the activation of brain-derived neurotrophic factor and its downstream signaling pathway.^[44–46]^ Ryffel et al suggested that protection of dopamine neuronal degeneration by acupuncture was due to enhancement of cyclophilin A (CypA) levels. Based on recently published studies, CypA is ubiquitously expressed in the brain^[46]^ and is predominantly localized in neurons.^[47]^ Another study shows that acupuncture possesses the ability to ameliorate mutant α-syn-induced motor abnormalities. This ability may be due to the that EA enhances both anti-inflammatory and antioxidant activities and suppresses aberrant glial activation in the diseased sites of brains.^[48]^

In a word, acupuncture treatments in animal experiments have generated valuable mechanistic insights into the pathology of Parkinson and have provided evidence that acupuncture therapy is neuroprotective and can increase various neuroprotective agents such as brain-derived neurotrophic factor, glial cell- derived neurotrophic factor, and cyclophilin A, enhancing both anti-inflammatory and antioxidant activities.

Traditional Chinese medicine has developed a relatively rich way, such as scalp acupuncture, body acupuncture, electroacupuncture, moxibustion, and acupuncture combined with other therapies and so on. The above methods have achieved remarkable results in improving patients’ cognition and activities of daily life.

It is worth noting that this study has some limitations. First of all, due to the low overall quality of the literature on acupuncture treatment of cognitive impairment after stroke, the final included literature is of low quality; in addition, there is little data related to adverse reactions and it is difficult to evaluate. Thirdly, most of the included subjects were aged between 45 and 70, while other age groups were underrepresented. Certain conditions that may be encountered in these age groups may lead to bias in the interpretation of the results. furthermore, none of the included studies considered the effect of gender. After that, further research in this area is needed. Due to the small amount of literature related to different types of acupuncture therapy, we did not conduct further subgroup analysis. Finally, it is suggested that a more scientific and rigorous research scheme should be designed in the future. It is hopeful to carry out the study through large samples and high-quality randomized controlled trials, to explore the differences between different types of acupuncture, and to provide a more scientific clinical basis for the popularization and application of acupuncture treatment for PSCI.

In conclusion, available evidence suggests that acupuncture combined with conventional therapy has a potential clinical advantage over conventional therapy in improving cognitive impairment after stroke. However, the differences between different types of acupuncture need to be clarified in more high-quality studies for further comparison.

## Author contributions

**Methodology:** Kang Xiong.

**Software:** Mei-Ling Zhang.

**Supervision:** Bin Wang.

**Writing – original draft:** Zhen-Zhi Wang, Zhen Sun.

**Writing – review & editing:** Zhen-Zhi Wang, Zhen Sun.

## References

[1] de Mendivil AO Alcal á-Galiano A Ochoa M. Brainstem stroke: anatomy, clinical and radiological findings. Semin Ultrasound CT MRI. 2013;34:131–41. 10.1053/j.sult.2013.01.004 23522778

[2] Claesson L Lindén T Skoog I. Cognitive impairment after stroke—impact on activities of daily living and costs of care for elderly people. The Göteborg 70+ Stroke Study. Cerebrovascular Dis (Basel, Switzerland). 2005;19:102–9. 10.1159/000082787 15608434

[3] Brainin M Tuomilehto J Heiss W-D. Post-stroke cognitive decline: an update and perspectives for clinical research. Eur J Neurol. 2015;22:229–38, e13. 25492161 10.1111/ene.12626

[4] Feigin Valery L Forouzanfar Mohammad H Krishnamurthi R. Global and regional burden of stroke during 1990-2010: findings from the global burden of disease study 2010. Lancet (London, England). 2014;54:245–54. 10.1016/s0140-6736(13)61953-4 PMC4181600 24449944

[5] Zhao NN Han JW Du YH. Research progress on mechanisms of bidirectional regulation of acupuncture. Zhongguo Zhen Jiu. 2021;41:1060–2. Chinese. 34491659 10.13703/j.0255-2930.20201021-0002

[6] Sze FK Wong E Or KK. Does acupuncture improve motor recovery after stroke? A meta-analysis of randomized controlled trials. Stroke. 2002;33:2604–19. 12411650 10.1161/01.str.0000035908.74261.c9

[7] Wu P Mills E Moher D. Acupuncture in poststroke rehabilitation: a systematic review and meta-analysis of randomized trials. Stroke. 2010;41:e171–9. 20167912 10.1161/STROKEAHA.109.573576

[8] Zhao XF Du Y Liu PG. Acupuncture for stroke: evidence of effectiveness, safety, and cost from systematic reviews. Top Stroke Rehabil. 2012;19:226–33. 22668677 10.1310/tsr1903-226

[9] Ye CH Ou CD Xu ZJ. Clinical observation on acupuncture treatment of post-stroke insomnia. Chinese J New Clin Med. 2013;6:665–7.

[10] Shah SH Engelhardt R Ovbiagele B. Patterns of complementary and alternative medicine use among United States stroke survivors. J Neurol Sci. 2008;271:180–5. 18485369 10.1016/j.jns.2008.04.014

[11] Liao CC Lin JG Tsai CC. An investigation of the use of traditional Chinese medicine in stroke patients in Taiwan. Evid Based Complement Alternat Med. 2012;2012:387164. 23304199 10.1155/2012/387164 PMC3530233

[12] Zhao ZQ. Neural mechanism underlying acupuncture analgesia. Prog Neurobiol. 2008;85:355–75. 18582529 10.1016/j.pneurobio.2008.05.004

[13] Ding X Yu J Yu T. Acupuncture regulates the aging-related changes in gene profile expression of the hippocampus in senescence-accelerated mouse (SAMP10). Neurosci Lett. 2006;399:11–6. 16516385 10.1016/j.neulet.2006.01.067

[14] Yu CC Wang J Ye SS. Preventive electroacupuncture Ameliorates D-Galactose-induced Alzheimer’s disease-like pathology and memory deficits probably via inhibition of GSK3β/mTOR signaling pathway. Evid Based Complement Alternat Med. 2020;2020:112–12. 10.1155/2020/1428752 PMC7195631 32382276

[15] Huang X Huang K Li Z. Electroacupuncture improves cognitive deficits and insulin resistance in an OLETF rat model of Al/D-gal induced aging model via the PI3K/Akt signaling pathway. Brain Res. 2020;1740:146834. article 32304687 10.1016/j.brainres.2020.146834

[16] Liang P Wang Z Qian T. Acupuncture stimulation of Taichong (Liv3) and Hegu (LI4) modulates the default mode network activity in Alzheimer’s disease. Am J Alzheimers Dis Other Demen. 2014;29:739–48. 24906968 10.1177/1533317514536600 PMC10852898

[17] Jia X Wang Z Huang F. A comparison of the Mini-Mental State Examination (MMSE) with the Montreal Cognitive Assessment (MoCA) for mild cognitive impairment screening in Chinese middle-aged and older population: a cross-sectional study. BMC Psychiatry. 2021;21:485. 34607584 10.1186/s12888-021-03495-6 PMC8489046

[18] Aguera-Ortiz LF Lopez-Alvarez J Del Nido-Varo L. Deterioro comportamental leve como antecedente de la demencia: presentacion de los criterios diagnosticos y de la version española de la escala MBI-C para su valoracion [Mild behavioural impairment as an antecedent of dementia: presentation of the diagnostic criteria and the Spanish version of the MBI-C scale for its evaluation]. Rev Neurol. 2017;65:327–334. Spanish. 28929475

[19] Almomani F Avi-Itzhak T Demeter N. Construct validity and internal consistency reliability of the Loewenstein occupational therapy cognitive assessment (LOTCA). BMC Psychiatry. 2018;18:184. 29890961 10.1186/s12888-018-1776-x PMC5996564

[20] Chen QS. Clinical observation of scalp acupuncture combined with repetitive transcranial magnetic stimulation on cognitive dysfunction in patients with stroke. Yunnan J Tradit Chin Med. 2020;41:60–2.

[21] Sha T Gao L Zheng JG. Clinical observation of acupuncture combined with medicine in the treatment of mild cognitive impairment after cerebral infarction. Inner Mongolia Tradit Chin Med. 2020;39:20–2.

[22] Yang L Tan J Chen LZ. Clinical observation of scalp acupuncture combined with hyperbaric oxygen in the treatment of cognitive impairment after stroke. Guide Tradit Chin Med. 2019;25:36–12.

[23] Xiang R Han LL Liu WG. Observation of the efficacy of scalp acupuncture combined with neck acupuncture and body acupuncture in the treatment of cognitive impairment after stroke. J Modern Integrated Tradit Chin Western Med. 2019;28:3391–4.

[24] Gao C Qi HB. A randomized controlled study of eye acupuncture combined with cognitive training in the treatment of vascular cognitive impairment after stroke. J Clin Acupuncture Moxibustion. 2019;35:12–5.

[25] Wu SC. Clinical observation of abdominal acupuncture combined with cognitive rehabilitation training in the treatment of mild cognitive impairment after stroke [D]. Guangxi Univ Traditional Chin Med. 2019.

[26] Liu L Xia WG Xu T. Clinical observation of scalp acupuncture combined with repetitive transcranial magnetic stimulation in the treatment of cognitive dysfunction after cerebral infarction. China Rehabilitation. 2019;34:123–126.

[27] Yang F Luo KT Yang XB. Clinical observation of Baihui long retaining Needle in the treatment of Cognitive dysfunction after apoplexy of Qi deficiency and Blood stasis. Chin Acupuncture Moxibustion. 2018;38:1151–1156. 10.13703/j.0255-2930.2018.11.004 30672194

[28] Han CY Wen WS Yao BL. Effect of cognitive training on cognitive function after stroke during scalp needle retention. Modern Pract Med. 2018;30:1592–1594.

[29] Teng X Zhou BF Gao LJ. Clinical study of eye-acupuncture in the treatment of cognitive impairment after stroke. Guide Tradit Chin Med. 2018;24:67–69.

[30] Zeng LL Zhou XL Huang Z. Clinical study of Jin three Needles combined with Cognitive Rehabilitation training in the treatment of Cognitive impairment in convalescent Stroke patients. Shandong J Tradit Chin Med. 2018;37:367–370.

[31] Jiang XM Zhong ZF Deng ZL. Clinical study of eye acupuncture combined with computer-assisted cognitive training on cognitive impairment in stroke. China Med Innovation. 2018;15:63–66.

[32] Liu M Geng P Liao HX. Observation on the efficacy of scalp acupuncture in the treatment of cognitive impairment after traumatic brain injury. Emergency Traditional Chin Med. 2017;26:106–8.

[33] Xue SL Gong DG Chen LC. Treatment of 30 cases of cognitive impairment after ischemic stroke by acupuncture with Zhi San acupuncture. Fujian Traditional Chin Med. 2016;47:20–21.

[34] Wu YX Wang ZC Wang ZX. To explore the effect of oxidative stress on cognitive impairment in the elderly from the perspective of deficiency, phlegm, and fire. Chin J Traditional Chin Med. 2019;37:2151–2154.

[35] Huang D. Analysis of the effect of rehabilitation training combined with Donepezil in the treatment of vascular cognitive impairment after stroke. Strait Pharm. 2018;30:150–151.

[36] Wang XH Meng CC Liu X. Effect of swallowing function evaluation scale combined with rehabilitation exercise on pulmonary infection in stroke patients with dysphagia. Chin J Hospital Epidemiol. 2017;27:4869–4872.

[37] Liu ZB Niu WM Yang XH. Effects of olfactory three on learning and memory function and activities of choline acetylase and acetylcholinesterase in hippocampus of senile dementia rats. Acupuncture Res. 2009;34:48–51.

[38] Rolland WB Lekic T Krafft PR. Fingolimod reduces cerebral lymphocyte infiltration in experimental models of rodent intracerebral hemorrhage. Exp Neurol. 2013;41:47–55. 10.1016/j.expneurol.2012.12.009 PMC3570752 23261767

[39] Feng Q Shang HJ Liu MQ. Effect of electroacupuncture on learning and memory ability and inflammatory factors in brain tissue of rats with vascular cognitive impairment. Chin J Traditional Chin Med Information. 2018;25:39–43.

[40] Zeng YH Bao JH Zhu M. Acupuncture treatment of mild cognitive impairment in subacute stroke: a randomized controlled study. Chin Acupuncture Moxibustion. 2015;35:979–982. 26790200

[41] Wang J Xing J He LJ. Research progress of acupuncture in the treatment of cognitive impairment after stroke. Global Tradit Chin Med. 2015;8:1140–1144.

[42] Li C Lu WH Xu JY. Research progress of acupuncture in the treatment of subarachnoid hemorrhage. J Shandong Univ Tradit Chin Med. 2012;36:81–82.

[43] Kim MW Chung YC Jung HC. Electroacupuncture enhances motor recovery performance with brain-derived neurotrophic factor expression in rats with cerebral infarction. Acupunct Med. 2012;30:222–6. 22729070 10.1136/acupmed-2011-010126

[44] Wang SJ Tan LH Liu JL. Effect of electroacupuncture at different acupoints on expression of cervico-spinal gdnf and bdnf and their receptor genes in neck-incision pain rats. Zhen Ci Yan Jiu. 2012;37:351–6. 23342772

[45] Hua C Liu QS. Effects of acupuncture on the expression of brain-derived neurotrophic factor in the ovariectomized rat fracture model. Zhongguo Zhen Jiu. 2009;29:303–8. 19565740

[46] Ryffel B Woerly G Greiner B. Distribution of the cyclosporine binding protein cyclophilin in human tissues. Immunology. 1991;72:399–404. 2026447 PMC1384402

[47] Göldner FM Patrick JW. Neuronal localization of the cyclophilin a protein in the adult rat brain. J Comp Neurol. 1996;372:283–93. 8863131 10.1002/(SICI)1096-9861(19960819)372:2<283::AID-CNE9>3.0.CO;2-#

[48] Deng J Lv E Yang J. Electroacupuncture remediates glial dysfunction and ameliorates neurodegeneration in the astrocytic α-synuclein mutant mouse model. J Neuroinflammation. 2015;12:103. 26016857 10.1186/s12974-015-0302-z PMC4449593

